# Kv1.1 potassium channel subunit deficiency alters ventricular arrhythmia susceptibility, contractility, and repolarization

**DOI:** 10.14814/phy2.14702

**Published:** 2021-01-11

**Authors:** Krystle Trosclair, Man Si, Megan Watts, Nicole M. Gautier, Niels Voigt, James Traylor, Miklós Bitay, Istvan Baczko, Dobromir Dobrev, Kathryn A. Hamilton, Md. Shenuarin Bhuiyan, Paari Dominic, Edward Glasscock

**Affiliations:** ^1^ Department of Cellular Biology & Anatomy Louisiana State University Health Sciences Center Shreveport LA USA; ^2^ Department of Internal Medicine Section of Cardiology Louisiana State University Health Sciences Center Shreveport LA USA; ^3^ Institute of Pharmacology and Toxicology University Medical Center Goettingen Goettingen Germany; ^4^ DZHK (German Center for Cardiovascular Research) Göttingen Germany; ^5^ Department of Pathology Louisiana State University Health Sciences Center Shreveport LA USA; ^6^ Department of Cardiac Surgery 2nd Department of Medicine and Cardiology Center University of Szeged Szeged Hungary; ^7^ Department of Pharmacology and Pharmacotherapy Interdisciplinary Excellence Centre University of Szeged Szeged Hungary; ^8^ Institute of Pharmacology West German Heart and Vascular Center University Duisburg‐Essen Essen Germany; ^9^ Department of Biological Sciences Southern Methodist University Dallas TX USA

**Keywords:** action potential, arrhythmia, contractility, K‐channel, repolarization

## Abstract

Epilepsy‐associated Kv1.1 voltage‐gated potassium channel subunits encoded by the *Kcna1* gene have traditionally been considered absent in heart, but recent studies reveal they are expressed in cardiomyocytes where they could regulate intrinsic cardiac electrophysiology. Although Kv1.1 now has a demonstrated functional role in atria, its role in the ventricles has never been investigated. In this work, electrophysiological, histological, and gene expression approaches were used to explore the consequences of Kv1.1 deficiency in the ventricles of *Kcna1* knockout (KO) mice at the organ, cellular, and molecular levels to determine whether the absence of Kv1.1 leads to ventricular dysfunction that increases the risk of premature or sudden death. When subjected to intracardiac pacing, KO mice showed normal baseline susceptibility to inducible ventricular arrhythmias (VA) but resistance to VA under conditions of sympathetic challenge with isoproterenol. Echocardiography revealed cardiac contractile dysfunction manifesting as decreased ejection fraction and fractional shortening. In whole‐cell patch‐clamp recordings, KO ventricular cardiomyocytes exhibited action potential prolongation indicative of impaired repolarization. Imaging, histological, and transcript analyses showed no evidence of structural or channel gene expression remodeling, suggesting that the observed deficits are likely electrogenic due to Kv1.1 deficiency. Immunoblots of patient heart samples detected the presence of Kv1.1 at relatively high levels, implying that Kv1.1 contributes to human cardiac electrophysiology. Taken together, this work describes an important functional role for Kv1.1 in ventricles where its absence causes repolarization and contractility deficits but reduced susceptibility to arrhythmia under conditions of sympathetic drive.

## Graphical abstract

The functions of Kv1.1 channels in ventricles were explored for the first time revealing that Kv1.1‐deficient mice exhibit resistance to pacing‐induced arrhythmias with isoproterenol, echocardiographic contractility deficits, and underlying action potential prolongation without evidence of structural or channel expression remodeling. Immunoblots of cardiac tissue from patients revealed Kv1.1 protein in human ventricles, suggesting potential involvement in human health and disease. These findings demonstrate new roles for Kv1.1 in the ventricles where it influences arrhythmia susceptibility, contractility, and repolarization.

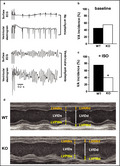

## 1 INTRODUCTION

Potassium ion channels are a numerous and diverse set of membrane proteins in the heart that are critical for mediating the repolarization of cardiac action potentials. Inherited or acquired potassium channelopathies can impair cardiac repolarization leading to common arrhythmias such as atrial fibrillation and long QT syndrome, which can increase the risk of mortality (Grant, 2009; Heijman et al., 2014; Nakano & Shimizu, 2016; Wilde & Bezzina, 2005). Nearly one‐third of sudden unexplained death cases in children are associated with variants in genes encoding cardiac ion channels, especially those that control cardiac repolarization (Campuzano et al., 2014). Voltage‐gated Kv1.1 potassium channel α‐subunits, which are associated with sudden unexpected death in epilepsy (SUDEP) in mice, have recently come to light as important regulators of cardiac repolarization and arrhythmia susceptibility in atria (Glasscock et al., 2015; Si et al., 2019). Following the discovery of Kv1.1 expression in mouse cardiomyocytes, electrophysiology studies in mice demonstrated that the lack of Kv1.1 channel subunits impairs repolarization leading to prolonged atrial action potentials and significantly increased risk of inducible atrial fibrillation (Glasscock et al., 2015; Si et al., 2019). Subsequent patient studies showed that human atrial cells also express Kv1.1 subunits and their associated currents, which are both augmented in chronic atrial fibrillation, suggesting a potential contribution by Kv1.1 channel remodeling (Glasscock et al., 2015). Previous studies of cardiac Kv1.1 have primarily focused on the atria, but Kv1.1 transcripts and protein are also detectable at low levels in mouse ventricular cardiomyocytes where their potential functional contribution remains unknown (Glasscock et al., 2015).

Kv1.1 subunits are best known for their roles in the nervous system where their dysfunction or absence leads to neuronal hyperexcitability and neurological disease (Glasscock, 2019; Paulhus et al., 2020). Kv1.1 exhibits broad expression in the brain with predominant localization in axons where they control action potential morphology and firing properties (Jan & Jan, 2012; Wang et al., 1994). In humans, *KCNA1* mutations cause the movement disorder episodic ataxia type 1 (EA1), as well as epilepsy, which is characterized by spontaneous recurrent seizures (Paulhus et al., 2020). Mice lacking Kv1.1 due to global *Kcna1* gene knockout (KO; i.e., *Kcna1* ^–/–^) exhibit spontaneous seizures with cardiorespiratory dysfunction, brain‐driven autonomic cardiac abnormalities, and premature seizure‐related death, making them a frequently used model for exploring neuro‐cardio‐respiratory mechanisms associated with SUDEP risk (Dhaibar et al., 2019; Glasscock et al., 2010; Hutson et al., 2020; Iyer et al., 2020; Moore et al., 2014; Simeone et al., 2018). Mice with neuron‐specific conditional knockout (cKO) of *Kcna1* exhibit an ameliorated premature death phenotype, living significantly longer than global knockouts (Trosclair et al., 2020). Thus, although Kv1.1 deficiency in neurons alone is sufficient for premature mortality, the preservation of cardiac Kv1.1 channels in cKO mice may provide an intrinsic cardioprotective role reducing the risk of seizure‐related death.

In this work, electrophysiological, histological, and gene expression approaches were used to explore the role of ventricular Kv1.1 channels at the organ, cellular, and molecular levels for the first time. Specifically, this study examined the hypothesis that Kv1.1 channels are functionally expressed in the ventricles where their absence leads to ventricular dysfunction that increases the risk of premature death. To test the consequences of Kv1.1 deficiency, in vivo intracardiac pacing and echocardiography were performed in global *Kcna1* KO mice to measure ventricular arrhythmia susceptibility and myocardial contractility. Whole‐cell patch‐clamp recordings were then performed on isolated mouse ventricular cardiomyocytes to examine the effects of genetic and pharmacological Kv1.1 ablation. Finally, Masson's trichrome histology and quantitative polymerase chain reaction (qPCR) analyses were used to evaluate the presence of deleterious structural and channel expression remodeling in Kv1.1‐deficient ventricles. Our findings reveal alterations in ventricular arrhythmia susceptibility, contractility, and repolarization associated with the absence of Kv1.1, providing the first evidence that Kv1.1 is required for normal ventricular function.

## 2 MATERIALS AND METHODS

### 2.1 Animals and Genotyping

*Kcna1* ^−/−^ knockout (KO) mice and wild‐type (WT) siblings were used for experiments. KO mice carry null alleles of the *Kcna1* gene (chromosome 6) resulting from the targeted deletion of the open reading frame, as previously described (Smart et al., 1998). The mice were bred and maintained on a Black Swiss (Tac:N:NIHS‐BC) genetic background. Animals were housed at 22°C, fed ad libitum, and maintained on a 12:12‐h light‐dark cycle. All procedures were performed in accordance with the guidelines of the National Institutes of Health (NIH), as approved by the Institutional Animal Care and Use Committee of the Louisiana State University Health Shreveport. For genotyping, genomic DNA was isolated by the enzymatic digestion of tail clips using Direct‐PCR Lysis Reagent (Viagen Biotech, Los Angeles, CA). The genotypes of *Kcna1* mice were determined by performing the PCR amplification of genomic DNA using allele‐specific primers: a KO‐specific primer (5'‐CCTTCTATCGCCTTCTTGACG‐3'), a WT‐specific primer (5'‐GCCTCTGACAGTGACCTCAGC‐3'), and a common primer (5'‐GCTTCAGGTTCGCCACTCCCC‐3'). The PCR yielded amplicons of ~337 bp for the WT allele and ~475 bp for the KO allele.

### 2.2 Electrocardiography (ECG) and intracardiac electrophysiology

In vivo pacing studies were performed using age‐ and sex‐matched 4‐month‐old KO and WT mice of both sexes (for WT: *n* = 29, including 9 males, 20 females; for KO: *n* = 20, including 7 males, 13 females). Mice were anesthetized with isoflurane (2% for induction and 1.5% for maintenance of anesthesia; Apollo Tech 3 Vaporizer; Norvap) and placed in a supine position with limbs taped onto surface electrocardiogram (ECG) electrodes of a temperature‐controlled procedure platform (Rodent Surgical Monitor, Indus Instruments, USA) which maintained core body temperatures at 37.0 ± 0.5°C. A Millar 1.1F octapolar EP catheter (EPR‐800; Millar Instruments) was inserted via an incision in the internal right jugular vein. The catheter was advanced to the right atrium and ventricle using electrogram guidance and pacing capture to verify intracardiac position. A computer‐based data acquisition system (PowerLab 16/30; ADI Instruments) was used to record a 4‐lead body surface ECG and up to six intracardiac bipolar electrograms (LabChart Pro software, version 7; AD Instruments). To induce ventricular arrhythmias, programmed electrical stimulation (PES) was performed using 2‐ms current pulses at 400 µA delivered by an external stimulator (STG‐3008 FA; Multi Channel Systems). As done previously (Berul et al., 2001), a burst pacing protocol with eight 50‐msec and four 30‐ms cycle length train episodes was used. This sequence was repeated twenty times every 3 s for a total of 70.4 s of the stimulation time. Ventricular arrhythmia (VA) was defined as a sequence of rapid spontaneous ventricular depolarizations that lasted >500 ms in response to PES. Each animal underwent three PES trials and was considered to be VA‐positive if VA was detected during any of the three trials. Following initial baseline PES trials, the β‐adrenergic receptor agonist isoproterenol hydrochloride (ISO; Sigma‐Aldrich, St. Louis, MO) was administered by intraperitoneal injection (4 mg/kg) to heighten ventricular susceptibility to arrhythmias, as done in human studies (Meester et al., 1997), and PES protocols were repeated. All VAs observed in the study were self‐terminating. All measurements were obtained by an observer blinded to genotype.

### 2.3 Echocardiography

Echocardiograms were obtained from isoflurane‐anesthetized female KO and WT mice approximately 6–8 months of age (*n* = 9–13 per genotype) using a VisualSonics Vevo 3100 Imaging System with a 30‐MHz transducer to assess cardiac functional parameters as described previously (Abdullah et al., 2018, 2019; Alam et al., 2018). Only female mice were analyzed for echocardiography because no age‐matched KO males were available at the time. Two‐dimensional directed M‐mode transthoracic echocardiographic images of the parasternal short axis were recorded to measure and compare the following parameters between genotypes: left ventricular internal diameter during systole (LVIDs); left ventricular internal diameter during diastole (LVIDd); left ventricular anterior and poster wall thickness during systole (LVAWs and LVPWs, respectively); and left ventricular anterior and poster wall thickness during diastole (LVAWd and LVPWd, respectively). These parameters were then used by an observer blinded to genotype to calculate stroke volume, cardiac output, ejection fraction fractional shortening, LV mass, and corrected LV mass using standard formulas (Gao et al., 2011).

### 2.4 Isolation of mouse ventricular myocytes

Ventricular myocytes were enzymatically isolated from hearts of age‐matched male and female KO and WT mice (ages 6–8 weeks). Briefly, mice were intraperitoneally injected with 5000 U/kg heparin (Sigma‐Aldrich, St. Louis, MO) and euthanized by cervical dislocation. The heart was quickly removed and mounted on a Langendorff apparatus followed by 3‐min retrograde perfusion with oxygenated (100% O_2_) Ca^2+^‐free Tyrode's solution containing (in mmol/l): 140 NaCl, 5.4 KCl, 0.5 MgCl_2_, 10 glucose, and 10 HEPES (pH 7.4; 37°C). Hearts were then perfused with the same Tyrode's solution but containing Liberase TH enzymes (0.025 mg/ml; Sigma‐Aldrich) and bovine serum albumin (BSA; 1 mg/ml; Sigma‐Aldrich). Left ventricular tissue was then removed and minced, and ventricular myocytes were dispersed in KB solution containing (in mmol/l): 80 KOH, 40 KCl, 25 KH_2_PO_4_, 3 MgSO_4_, 50 glutamic acid, 20 taurine, 1 EGTA, 10 glucose, and 10 HEPES (pH 7.2 with KOH; 20–22°C). Cells were stored at room temperature (20–22°C) for at least 1 h before use. All chemicals used to make the solutions for cell isolations were obtained from Sigma‐Aldrich.

### 2.5 Whole‐cell patch‐clamp recordings

Whole‐cell patch‐clamp recordings were obtained from the isolated ventricular myocytes at 37°C. Borosilicate glass pipette (Warner Instruments, Hamden, CT) microelectrodes were used with tip resistances of 2–4 MΩ when filled with pipette solution. Electrodes were connected to a MultiClamp 700B microelectrode amplifier equipped with a CV‐7B head stage (Axon Instruments, Molecular Devices, San Jose, CA). Electrical signals were sampled at 4 kHz and digitized with an Axon analog/digital converter (Digidata 1440A). Data acquisition and analysis were performed using Clampfit software (version 10.3, Axon Instruments, Molecular Devices). For current‐clamp recordings, action potentials were evoked by electrical stimulation with 1‐ms, 2‐nA current pulses at a frequency of 1 Hz. The bath solution contained (in mmol/l): 126 NaCl, 5.4 KCl, 1.8 CaCl_2_, 1.0 MgCl_2_, 20 HEPES, and 11 glucose (pH = 7.4 with NaOH). The pipette solution contained (in mmol/l): 90 K‐aspartate, 30 KCl, 10 NaCl, 5.5. glucose, 1.0 MgCl_2_, 10 EGTA, 4.0 Na‐GTP, and 10 HEPES (pH = 7.2 with KOH). Using the JPCalc application in the Clampex software (Molecular Devices), the liquid junction potential was estimated to be 13.4 mV at 37°C. Resting membrane potential was calculated by correcting for the liquid junction potential. The series resistance was <10 MΩ for all measurements included in the study and it was not compensated. Dendrotoxin‐K (10 nM; Sigma‐Aldrich) was used to selectively block Kv1.1 channels, as done previously (Si et al., 2019). All chemicals used to make the bath and pipette solutions for recordings were obtained from Sigma‐Aldrich.

### 2.6 Histology

Whole hearts from age‐matched 4‐ to 6‐month‐old mice (*n* = 5 per genotype; for WT: 5 females; for KO: 3 females, 2 males) were excised following euthanasia by isoflurane overdose and then rinsed briefly in phosphate‐buffered saline (PBS), fixed in 10% neutral buffered formalin for 24 h, and transferred to 50% ethanol. Hearts were then embedded in paraffin, serial sectioned (5 μm) in the longitudinal (long axial) plane, mounted onto slides, and stained with Masson's trichrome. Microscopic cardiac pathology was determined by a board‐certified and licensed expert pathologist (Dr. J. Traylor) who was blinded to genotype.

### 2.7 Quantitative PCR analyses

Following euthanasia by cervical dislocation, a portion of the left ventricle of age‐ and sex‐matched 6‐ to 8‐week‐old WT and KO mice (for WT: *n* = 13, including 7 males, 6 females; for KO: *n* = 10, including 5 males, 5 females) was quickly harvested and homogenized in ice‐cold TRI reagent (Zymo Research, Irvine, CA). The tissue was carefully selected from the outer wall of the left ventricle equidistant from the base and apex and approximately 1 mm^3^ in volume. Total RNA was extracted using the PureLink RNA Mini Kit (Thermo Fisher, Waltham, MA). Genomic DNA was eliminated using the DNA‐free DNA Removal Kit (Thermo Fisher). The quantity of total RNA was measured using a NanoDrop 1000 spectrophotometer (Thermo Fisher) and quality was confirmed using an RNA ScreenTape assay on a 2200 TapeStation system (Agilent, Santa Clara, CA). The resulting RNA integrity number was used to estimate total RNA integrity and only samples with scores >8.0 were used for experiments. The RNA samples (375 ng) were converted to first‐strand cDNA using the iScript Advanced cDNA Kit for reverse transcriptase (RT)‐quantitative PCR (qPCR) with oligo(dT) primers (Bio‐Rad, Hercules, CA). qPCR of first‐strand cDNA was performed with TaqMan gene expression assays (Thermo Fisher) that were designed and preoptimized by Thermo Fisher for the detection of *Kcna4* (Mm00445241_s1), *Kcna5* (Mm00524346_s1), *Kcnb1* (Mm00492791_ m1), *Kcnd2* (Mm01161732_m1), *Kcnh2* (Mm00465377_mH), *Kcnj2* (Mm00434616_m1), *Kcnj3* (Mm00434618_m1), *Kcnq1* (Mm00434640_ m1), *Scn5a* (Mm01342518_m1), and *Cacna1c* (Mm01188822_m1). qPCR experiments were performed using a 1:15 cDNA dilution, which was determined to be the optimal concentration by analyzing qPCR amplification across a five‐point cDNA dilution series to generate a standard curve. Individual PCR reactions were performed in triplicate using cDNA and TaqMan Gene Expression Master Mix (Thermo Fisher) on a CFX96 Fast Real‐Time PCR System (Bio‐Rad). No template and no reverse transcriptase (–RT) reactions were included as negative controls to verify the absence of contamination leading to unwanted PCR amplification and detection. Reactions were normalized to the amplification threshold cycle (C_T_) of the housekeeping gene hypoxanthine phosphoribosyltransferase 1 (*Hprt1*; Mm03024075_m1). Relative mRNA expression was calculated as normalized values by use of the 2^−ΔΔCT^ formula.

### 2.8 Human protein measurements

Samples of right atrial (*n* = 6) and left ventricular (*n* = 3) tissue from healthy control hearts were obtained from six male donors between the ages of 44–59 years old. Hearts were obtained from general organ donors whose undiseased hearts were explanted to obtain pulmonary and aortic valves for transplant surgery. Before cardiac explantation, organ donors did not receive medication other than dobutamine and plasma expanders. The cardioplegic solution used was Custodiol. All samples were frozen in liquid nitrogen and stored at −80°C. Sample collection and the experimental protocols were approved by the Scientific Board at the Hungarian Ministry of Health (ETT‐TUKEB: 4991‐0/2010‐1018EKU) and performed in accordance with the Declaration of Helsinki. Each patient gave written informed consent. Protein levels of *KCNA1* (1:1000; Abcam, Cambridge, MA) were quantified by Western blotting and normalized to GAPDH (1:200,000; HyTest, Turku, Finland). Peroxidase‐conjugated goat anti‐rabbit (1:5,000; Sigma‐Aldrich, St. Louis, MO) and goat anti‐mouse (1:50,000; Sigma‐Aldrich) were used as secondary antibodies and visualized by chemifluorescencea (GE Healthcare, Chalfont St. Giles, UK). AIDA Image Analyzer Software (raytest, Straubenhardt, Germany) was used for analysis.

### 2.9 Statistical analysis

All data are expressed as means ± standard deviation. For whole‐cell patch‐clamp electrophysiology data, the sample sizes (n) indicate the numbers of cells recorded and mice (i.e., total number of cells recorded/total number of mice used). Statistical analyses were performed using Prism for Windows (version 9; GraphPad Software, La Jolla, CA) and OriginPro (version 7.5, OriginLab, Northampton, MA). The D’Agostino‐Pearson test was used to test the normality of all electrophysiology and echocardiography data and the distributions were found to be normal in all cases except for the APD_50_ values with DTX‐K in WT mice and the echocardiographic measurements of LVPWd in WT mice and LVAWd in KO mice. For comparisons involving two groups, either paired or unpaired two‐tailed Student's *t*‐tests were employed as appropriate. The action potential duration data were also analyzed using nested (i.e., hierarchical) *t*‐tests and no significant differences were identified between animals of the same genotype.

## 3 RESULTS

### 3.1 Kv1.1 deficiency alters ventricular repolarization and arrhythmia susceptibility

**FIGURE 1:**
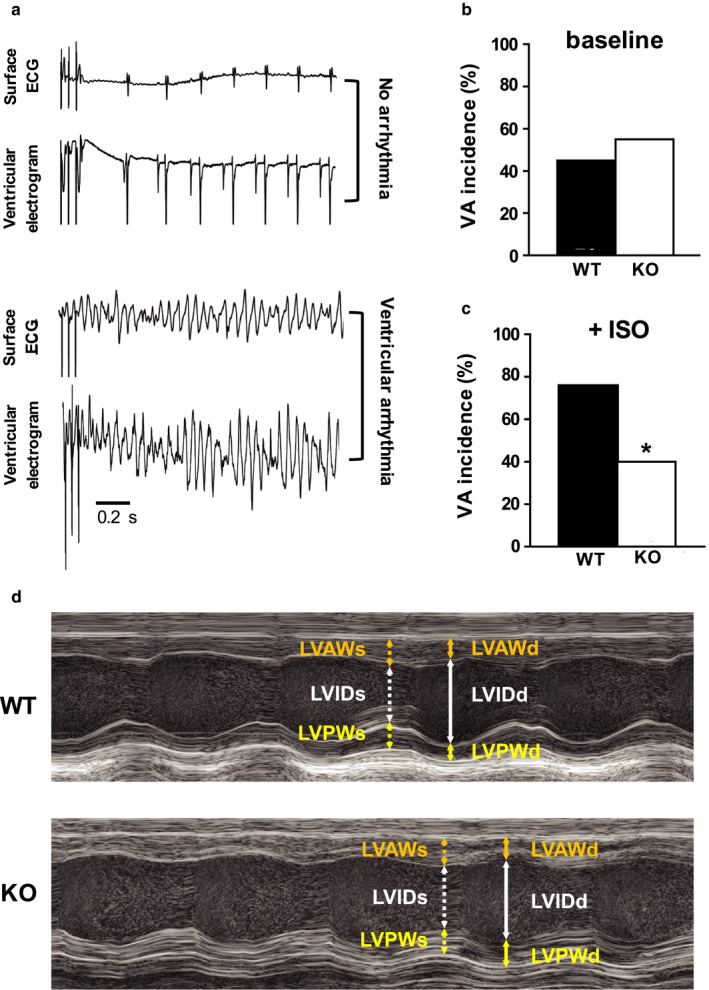
Alterations of ventricular electrophysiology and function in *Kcna1* ^⎯/⎯^ mice. (A) Representative surface ECG (lead I) and ventricular electrogram traces after pacing stimulation exhibiting no arrhythmia (top two traces) versus arrhythmia (bottom two traces) responses. (B‐C) Incidence of pacing‐induced ventricular arrhythmia (VA) (B) at baseline and (C) following isoproterenol (ISO) administration (4 mg/kg, i.p.) in WT (*n* = 29) and KO mice (*n* = 20). (D) Examples of M‐mode echocardiography images from WT and KO mice showing increased left ventricular internal diameter during systole (LVIDs; dotted white arrow) in KO animals that leads to decreased ejection fraction and fractional shortening. The following parameters are also indicated: left ventricular internal diameter during diastole (LVIDd; solid white arrow); left ventricular anterior and poster wall thickness during systole [(LVAWs; dotted orange arrow) and (LVPWs; dotted yellow arrow), respectively]; and left ventricular anterior and poster wall thickness during diastole [(LVAWd; solid orange arrow) and (LVPWd; solid yellow arrow), respectively]. *, *p* < 0.05 (Fisher's exact test)

To determine whether Kv1.1 deficiency leads to altered ventricular arrhythmia (VA) susceptibility, intracardiac programmed electrical stimulation (PES) was performed on adult WT (*n* = 29) and *Kcna1*‐null (KO) animals (*n* = 20), and ventricular responses were monitored and recorded using surface ECG and ventricular electrograms (Figure 1A‐C). In response to PES, the incidence of inducible VA was similar between genotypes: 45% of WT animals were VA‐positive at baseline versus 55% of KO animals (*p* = 0.57, Fisher's exact test; Figure 1B). Following the administration of the β‐adrenergic agonist, isoproterenol, which usually enhances susceptibility to VA (Meester et al., 1997), WT animals exhibited a significantly higher incidence of pacing‐induced VA (76%) compared to KO animals (40%; *p* = 0.017, Fisher's exact test; Figure 1C). These experiments provide evidence that the genetic deletion of Kv1.1 subunits decreases ventricular arrhythmia susceptibility under conditions of sympathetic challenge. Whether changes in VA incidence were associated with changes in the QT interval could not be ascertained since the T wave was not reliably identifiable in the surface ECG recordings.

### 3.2 Kv1.1 deficiency impairs ventricular contractility and efficiency

Echocardiographic measurements revealed significantly decreased ventricular function in KO hearts compared to WT hearts (Figure 1D). Specifically, the left ventricles (LVs) of KO mice exhibited significantly decreased stroke volume (−25%; *p* = 0.033, unpaired *t*‐test), ejection fraction (−17%; *p* = 0.006, unpaired *t*‐test), cardiac output (−26%; *p* = 0.001, unpaired *t*‐test), and fractional shortening (−32%; *p* = 0.005, unpaired *t*‐test), indicating impaired cardiac contractility and efficiency. However, measures of LV cavity dimensions and wall thickness in KO hearts were similar to WT, suggesting that the functional deficits in KO hearts were not associated with structural remodeling (Table 1).

**TABLE 1:** Echocardiographic measurements in WT and KO mice

	WT (*n* = 11)	KO (*n* = 9)	*p*‐value
HR (bpm)	427 ± 56	385 ± 54	0.108
SV (mm^3^)	35 ± 8	26 ± 9	0.033*
EF (%)	74 ± 11	61 ± 7	0.006**
FS (%)	44 ± 10	32 ± 5	0.005**
CO (ml/min)	15 ± 2	10 ± 3	0.001**
LV mass (mg)	111 ± 23	122 ± 37	0.440
LV mass corr (mg)	89 ± 19	98 ± 29	0.440
LVAWs (mm)	1.6 ± 0.4	1.4 ± 0.5	0.380
LVAWd (mm)	1.2 ± 0.5	1.1 ± 0.4	0.616
LVPWs (mm)	1.3 ± 0.3	1.1 ± 0.4	0.733
LVPWd (mm)	0.9 ± 0.3	1.0 ± 0.3	0.545
LVIDs (mm)	3.4 ± 0.8	3.4 ± 0.5	0.381
LVIDd (mm)	2.0 ± 0.9	2.3 ± 0.5	0.358

Data are expressed as mean ± standard deviation. 2‐tailed unpaired Student’s *t*‐test was used to compare intragroup differences.

Abbreviations: HR, heart rate; SV, stroke volume; EF, ejection fraction; FS, fractional shortening; CO, cardiac output; LV Mass, left ventricular mass; LV Mass Corr, corrected left ventricular mass; LVAWs, left ventricular anterior wall thickness during systole; LVAWd, left ventricular anterior wall thickness during diastole; LVIDs, left ventricular internal diameter during systole; LVIDd, left ventricular internal diameter during diastole; LVPWs, left ventricular posterior wall thickness during systole; LVPWd, left ventricular posterior wall thickness during diastole; bpm, beats per minute.

* *p* < 0.05.

** *p* < 0.01.

### 3.3 Kv1.1 deficiency prolongs ventricular action potentials

**FIGURE 2:**
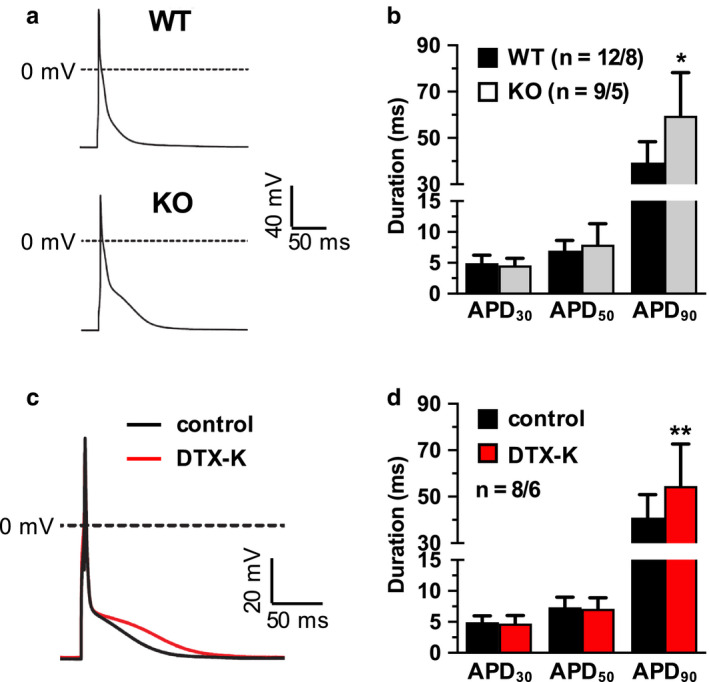
Effects of Kv1.1 deletion and inhibition on action potential morphology of ventricular cardiomyocytes. (A) Representative whole‐cell patch‐clamp action potential recordings from wild‐type (WT) and *Kcna1* ^⎯/⎯^ (KO) myocytes. (B) Average action potential duration (APD) in WT and KO cells at 30, 50, and 90% repolarization (APD_30_, APD_50_, and APD_90_, respectively) (C) Representative ventricular action potential recordings in a WT cell at baseline (black line) overlaid with the resulting action potential after the application of 10 nM DTX‐K (red line). (D) Average action potential duration (APD) in WT cells at APD_30_, APD_50_, and APD_90_ before (control) and after (DTX‐K) application of 10 nM DTX‐K. Sample numbers (n) indicate numbers of myocytes and mice. *, *p* < 0.05 (2‐tailed unpaired Student's *t*‐test); **, *p* < 0.01 (2‐tailed paired Student's *t*‐test).

To determine the functional effects of Kv1.1 deficiency on the ventricles at the cellular level, whole‐cell patch‐clamp recordings of action potentials were obtained from isolated WT and KO ventricular cardiomyocytes. In current‐clamp recordings, KO cells (*n* = 9) exhibited significantly prolonged action potential durations (+51%) compared to WT cells (*n* = 12) when measured at 90% repolarization (APD_90_), which corresponds to the late phase of the action potential (*p* = 0.0039, unpaired *t*‐test; Figure 2A,B). However, no significant differences were observed between genotypes for the APDs of the early (APD_30_; *p* = 0.50, unpaired *t*‐test) and middle phases (APD_50_; *p* = 0.39, unpaired *t*‐test). Blockade of Kv1.1 channels using the Kv1.1‐specific inhibitor dendrotoxin‐K (DTX‐K; 10 nM) caused 33% prolongation of APD_90_ in WT cells (*n* = 8; *p* = 0.037, paired *t*‐test; Figure 2C,D), but APD_30_ (*p* = 0.30, paired *t*‐test) and APD_50_ (*p* = 0.54, paired *t*‐test), were not significantly affected, mimicking the above observations in KO cells and demonstrating that both genetic and pharmacological inhibition of Kv1.1 subunits impairs ventricular repolarization. Importantly, previous recordings from mouse cardiomyocytes showed that DTX‐K has no effect on APD in KO animals, as expected since DTX‐K is specific for Kv channels containing Kv1.1 subunits (Si et al., 2019). Baseline resting membrane potential was not significantly different between WT (−97 ± 10 mV) and KO (−95 ± 6 mV) ventricular cardiomyocytes (*p* = 0.54, unpaired *t*‐test).

### 3.4 Kv1.1 deficiency does not cause structural or ion channel remodeling in ventricles

Since myocardial scarring, or fibrosis, is known to be associated with alterations in overall cardiac function (Morita et al., 2014), the hearts of KO mice were inspected for microscopic structural lesions, such as excessive collagen deposition and cell death (Figure 2B). Masson's trichrome staining of WT and KO ventricular tissue (*n* = 5 per genotype) revealed the presence of minor focal subendocardial fibrosis that was restricted to the papillary muscles, but this finding was present in both genotypes in 40% of hearts. Notably, KO hearts revealed no evidence of increased myocardial interstitial fibrosis in the ventricular wall, where it could have a detrimental impact on cardiac function. The absence of both microscopic and macroscopic (Figure 1D; Table 1) structural abnormalities in KO ventricles suggests that the alterations in heart function due to Kv1.1 deficiency are likely electrophysiological rather than structural in nature.

**FIGURE 3:**
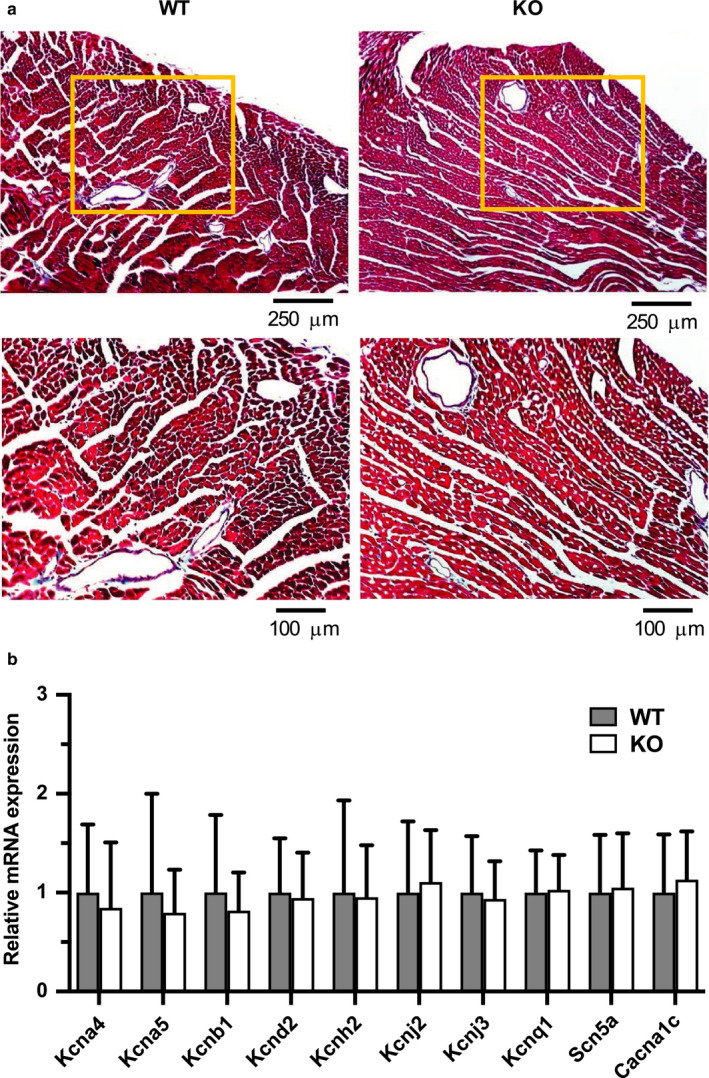
Absence of obvious structural and expression remodeling in *Kcna1* ^⎯/⎯^ mice. (A) Representative images of ventricular sections from WT (*n* = 5) and KO (*n* = 5) animals stained with Masson's trichrome to visualize fibrosis. Positive fibrosis staining appears light blue in color. The boxed regions in the upper panels are shown at higher magnification in the lower panels. (B) Relative mRNA expression profile of ion channel subunits important for action potential generation, as measured in left ventricular tissue from WT (*n* = 13) and KO (*n* = 10) animals. Expression levels were normalized to *Hprt1* as a reference gene.

To test whether *Kcna1* deletion leads to changes in the expression of other ventricular ion channels which could underlie the electrophysiological phenotypes observed, quantitative RT‐PCR (qPCR) was performed to measure mRNA transcript levels of other prominent ion channels that contribute to action potential morphology and repolarization (Figure 3B). The following genes (with their associated protein and current) were measured: *Kcna4* (Kv1.4; I_to,s_); *Kcna5* (Kv1.5; I_K,slow1_); *Kcnb1* (Kv2.1; I_K,slow2_); *Kcnd2* (Kv4.2; I_to,f_); *Kcnh2* (Kv11.1/mERG; I_Kr_); *Kcnj2* (Kir2.1; I_K1_); *Kcnj3* (Kir3.1; I_K,ACh_); *Kcnq1* (Kv7.1; I_ss_); *Scn5a* (Nav1.5; I_Na_); and *Cacna1c* (Cav1.2; I_Ca,L_). qPCR comparisons revealed no significant remodeling of any single ventricular ion channel gene due to *Kcna1* deletion, suggesting that the absence of Kv1.1 channels, and not expression remodeling of other channels, may be the primary defect underlying the observed electrophysiological alterations in the ventricles of KO hearts. One inherent limitation of our measurements was the presence of non‐myocyte cell types in our ventricular tissue samples which could contribute to gene expression. In addition, Kv1.1 deficiency could also influence cardiac physiology by inducing changes in other channels at the levels of protein expression, post‐translational regulation, or subcellular regulation.

### 3.5 Kv1.1 protein is present in human ventricles

**FIGURE 4:**
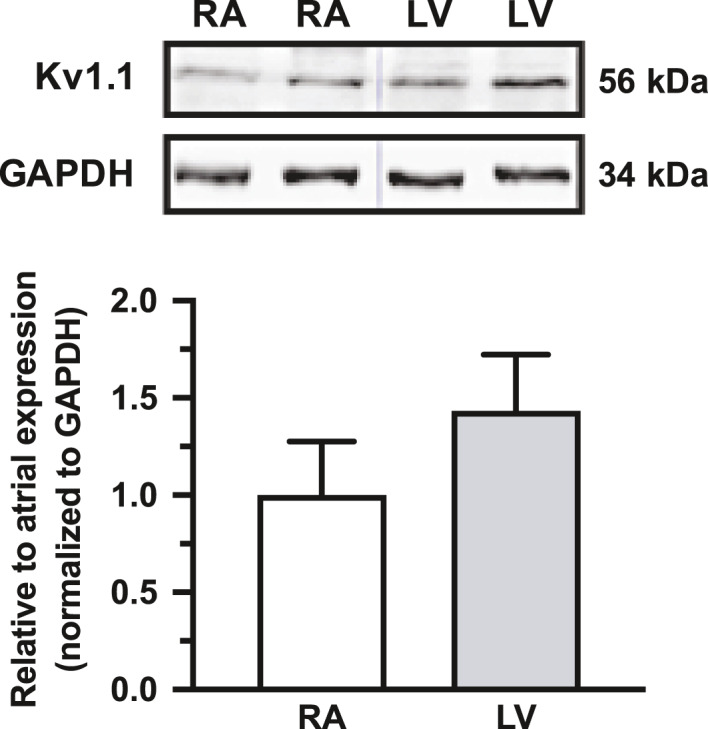
Kv1.1 protein is present in both human atria and ventricles. Representative western blot of Kv1.1 protein in two human right atrial (RA) and left ventricular (LV) samples with corresponding densitometric quantification (mean ± standard deviation) of protein levels normalized to GAPDH (loading control) and expressed relative to atrial Kv1.1 expression

To determine if ventricular Kv1.1 subunits may have potential functional significance in patients, human ventricle samples (*n* = 3) were examined for the presence of Kv1.1 protein and the levels compared to atrial samples (*n* = 6). Using immunoblotting, Kv1.1 was detected in human ventricles for the first time. Interestingly, protein quantification showed that Kv1.1 was not only present but abundant in human ventricles at levels similar to atria (*p* = 0.064, 2‐tailed unpaired *t*‐test), which is in contrast to mice which exhibit higher levels in atria (Figure 4) (Glasscock et al., 2015). However, because these experiments were performed in tissue samples and not isolated myocytes, a contribution by other non‐myocyte cell types cannot be excluded.

## 4 DISCUSSION

This study reveals new functional roles for the recently discovered cardiac ion channel Kv1.1 in regulating ventricular arrhythmia susceptibility, contractility, and repolarization. By several electrophysiological approaches, this work demonstrates that the genetic ablation of *Kcna1* impacts the function of the ventricles at the organ and cellular levels. Although KO mice exhibited normal susceptibility to inducible VA at baseline, they showed resistance to VA relative to WT mice when subjected to sympathetic challenge with isoproterenol. Echocardiography revealed additional evidence of ventricular dysfunction, including deficits in ejection fraction and fractional shortening. At the cellular level, APD prolongation was evident in recordings of isolated ventricular cardiomyocytes from KO mice. Notably, no evidence of structural or ion channel gene expression remodeling was observed in ventricles from KO mice, suggesting that the functional consequences of Kv1.1 deficiency are electrical in origin. Finally, protein analysis of human cardiac tissue revealed that Kv1.1 is not only present in the ventricles but that it is also expressed at levels comparable to atrial Kv1.1, further supporting the importance of the future exploration of the role of Kv1.1 in normal and pathological ventricular electrophysiology.

Because *Kcna1* is dually expressed in both heart and brain, a single gene mutation can theoretically cause altered excitability of both organ systems and thereby result in a “double hit” on cardiac function that could potentially increase the risk of sudden death, especially when seizures occur (Glasscock, 2014; Goldman, 2015; Goldman et al., 2016). Previous studies have shown that Kv1.1 deficiency can promote cardiac abnormalities by brain‐mediated mechanisms via autonomic dysregulation that can lead to SUDEP (Glasscock et al., 2010, 2012; Mishra et al., 2017; Moore et al., 2014). In addition, neuron‐specific deletion of *Kcna1* in mice causes a milder SUDEP phenotype than global gene deletion, suggesting that Kv1.1 deficiency in the heart combines with Kv1.1 deficiency in the brain to augment risk (Trosclair et al., 2020). In this study, the absence of Kv1.1 in the heart did not render it more susceptible to sympathetic‐mediated arrhythmogenic ventricular dysfunction since isoproterenol administration did not augment VA susceptibility, suggesting that Kv1.1 KO mice may be resistant to catecholaminergic effects of seizures on the heart. However, as demonstrated previously in canine cardiac recordings, the administration of catecholamines does not exactly mimic the pro‐fibrillatory effects of the intrinsic activation of the sympathetic nervous system by direct or reflex stimulation (Han & Moe, 1964). Furthermore, the mode of sympathetic stimulation, whether a sympathetic surge (such as a seizure) or high sympathetic tone (such as continuous β‐adrenergic stimulation), can have differential effects on arrhythmia susceptibility depending on the underlying genetics and pathophysiology (Liu et al., 2012). Thus, it remains to be determined whether the resistance of Kv1.1 KO mice to inducible VA in the presence of isoproterenol is indicative of an intrinsic cardioprotective effect of Kv1.1‐deficiency against spontaneous seizure‐related catecholamine surges. In addition, previous studies suggest that parasympathetic mechanisms may be of greater importance for SUDEP risk in this model since global Kv1.1 KO mice exhibit seizure‐associated bradycardia and asystole immediately preceding death, as well as prolonged survival with unilateral vagotomy (Glasscock et al., 2010; Moore et al., 2014). One possibility is that Kv1.1 KO mice exhibit underlying alterations in β‐adrenergic receptor expression or circulating catecholamine levels which could influence their isoproterenol response but this remains to be determined. Additional studies will be required to clarify the complex relationship between Kv1.1 deficiency in the heart and susceptibility to seizure‐related cardiac arrhythmias that could contribute to sudden death.

This study provides the first characterization of the role of Kv1.1 in the ventricles, allowing comparisons to be made between Kv1.1 expression and function in ventricular versus atrial tissues where Kv1.1 has already been examined. Although Kv1.1 is present at both the transcript and protein level in atrial and ventricular cardiomyocytes, quantitative PCR and immunocytochemistry studies indicate a higher abundance of Kv1.1 (up to ~10‐fold) in murine atrial cells compared to ventricular cells, suggesting possible differences in the functional importance of Kv1.1 in the two regions (Glasscock et al., 2015). While neither atrial nor ventricular tissues from Kv1.1 KO mice exhibit abnormal levels of interstitial fibrosis, the tissues show markedly different electrophysiological properties (Glasscock et al., 2015; Si et al., 2019). For example, Kv1.1 deficiency is arrhythmogenic in atria, but has no effect on arrhythmia susceptibility in the ventricles except under conditions of β‐adrenergic stimulation when it is apparently arrhythmia‐resistant (Glasscock et al., 2015). Additionally, Kv1.1 deficiency leads to ion channel transcript remodeling in the atria, but not in the ventricles (Si et al., 2019). At the cellular level, both atrial and ventricular Kv1.1‐deficient cardiomyocytes have prolonged action potentials, but their degree of prolongation is chamber‐specific. Atrial KO cells exhibit an average APD_90_ prolongation of ~100% compared to WT, whereas APD_90_ in ventricular KO cells was only ~50% longer (Si et al., 2019). Finally, in contrast to Kv1.1 expression patterns in mice, patient samples in the present study show similar levels of Kv1.1 protein in the ventricles and atria, suggesting the importance of Kv1.1 in human heart in both chambers. Taken together, these findings emphasize the need to further investigate regional differences in the role of Kv1.1 in the heart.

A potential limitation of this study for inferring cardiac mechanisms of SUDEP is that the ages of the animals used for in vivo measurements were beyond the time window when premature death most frequently occurs in this model. Our previous studies show that most Kv1.1 KO mice exhibit premature death between the ages of 2 to 7 weeks old (Trosclair et al., 2020). However, the mice used for the in vivo and histological experiments in this research ranged in age from approximately 17 to 34 weeks. Older mice were required in this study to address technical considerations, such as needing larger, older animals for venous catheter insertion in intracardiac pacing experiments and to increase the likelihood of detecting echocardiographic deficits, which tend to predominate in older animals. Another limitation of our echocardiographic analyses was that measurements were only performed in female mice of both genotypes due to a lack of availability of age‐matched male KO mice. Although the ages of the mice in our study tended to be older than the age window of maximal SUDEP risk in Kv1.1 KO mice, our findings still demonstrate the general cardiac effects of chronic Kv1.1 deficiency and the important requirement of Kv1.1 channels for normal heart function.

In summary, this work discloses a functional role for Kv1.1 subunits in the ventricles for the first time by demonstrating that *Kcna1* gene deletion in mice impairs both contractility and cardiac repolarization and alters arrhythmia susceptibility. Furthermore, the absence of significant underlying structural or channel remodeling implies these abnormalities are electrical in origin. The detectable levels of Kv1.1 in human ventricles suggest *KCNA1* could contribute to human ventricular pathology. However, given the lack of reported cardiac symptoms in patients with EA1 or epilepsy, the cardiac repolarization reserve may be capable of compensating for Kv1.1‐associated repolarization abnormalities. Alternatively, since traditionally Kv1.1 subunits have not been considered to have a role in the heart, the lack of identified Kv1.1‐associated cardiac deficits in patients could be due to ascertainment bias from patients not being clinically examined for cardiac dysfunction. As our understanding of the pathological mechanisms of inherited cardiac arrhythmias and SUDEP continues to evolve, it is important to consider the previously overlooked role of Kv1.1 in the ventricles and its potential impact on cardiac electrophysiology in health and disease.

## CONFLICT OF INTERESTS

None.

## Data Availability

The data that support the findings of this study are available from the corresponding author upon reasonable request.
